# Prognosemodelle zur Steuerung von intensivmedizinischen COVID-19-Kapazitäten in Deutschland

**DOI:** 10.1007/s00063-022-00903-x

**Published:** 2022-03-10

**Authors:** Marlon Grodd, Lukas Refisch, Fabian Lorenz, Martina Fischer, Matthäus Lottes, Maren Hackenberg, Clemens Kreutz, Linus Grabenhenrich, Harald Binder, Martin Wolkewitz

**Affiliations:** 1 Institut für Medizinische Biometrie und Statistik, Medizinische Fakultät und Universitätsklinikum, Albert-Ludwigs-Universität Freiburg, Freiburg, Deutschland; 2 Robert Koch-Institut, Berlin, Deutschland

**Keywords:** Pandemien, Intensivstationen, Bettenbelegung, Zuteilung von Ressourcen, Statistisches Modell, Pandemics, Intensive care units, Bed occupancy, Resource allocation, Statistical models

## Abstract

**Hintergrund:**

Zeitdynamische Prognosemodelle spielen eine zentrale Rolle zur Steuerung von intensivmedizinischen COVID-19-Kapazitäten im Pandemiegeschehen. Ein wichtiger Vorhersagewert (Prädiktor) für die zukünftige intensivmedizinische (ITS-)COVID-19-Bettenbelegungen ist die Anzahl der SARS-CoV-2-Neuinfektionen in der Bevölkerung, die wiederum stark von Schwankungen im Wochenverlauf, Meldeverzug, regionalen Unterschieden, Dunkelziffer, zeitabhängiger Ansteckungsrate, Impfungen, SARS-CoV-2-Virusvarianten sowie von nichtpharmazeutischen Eindämmungsmaßnahmen abhängt. Darüber hinaus wird die aktuelle und auch zukünftige COVID-ITS-Belegung maßgeblich von den intensivmedizinischen Entlassungs- und Sterberaten beeinflusst.

**Methode:**

Sowohl die Anzahl der SARS-CoV-2-Neuinfektionen in der Bevölkerung als auch die intensivmedizinischen COVID-19-Bettenbelegungen werden bundesweit flächendeckend erfasst. Diese Daten werden tagesaktuell mit epidemischen SEIR-Modellen aus gewöhnlichen Differenzialgleichungen und multiplen Regressionsmodellen statistisch analysiert.

**Ergebnisse:**

Die Prognoseergebnisse der unmittelbaren Entwicklung (20-Tage-Vorhersage) der ITS-Belegung durch COVID-19-Patienten*innen werden Entscheidungsträgern auf verschiedenen überregionalen Ebenen zur Verfügung gestellt.

**Schlussfolgerung:**

Die Prognosen werden der Entwicklung von betreibbaren intensivmedizinischen Bettenkapazitäten gegenübergestellt, um frühzeitig Kapazitätsengpässe zu erkennen und kurzfristig reaktive Handlungssteuerungen, wie etwa überregionale Verlegungen, zu ermöglichen.

## Abstract (en)

**Background:**

Time-series forecasting models play a central role in guiding intensive care coronavirus disease 2019 (COVID-19) bed capacity in a pandemic. A key predictor of future intensive care unit (ICU) COVID-19 bed occupancy is the number of new severe acute respiratory syndrome coronavirus 2 (SARS-CoV-2) infections in the general population, which in turn is highly associated with week-to-week variability, reporting delays, regional differences, number of unknown cases, time-dependent infection rates, vaccinations, SARS-CoV‑2 virus variants, and nonpharmaceutical containment measures. Furthermore, current and also future COVID ICU occupancy is significantly influenced by ICU discharge and mortality rates.

**Methods:**

Both the number of new SARS-CoV‑2 infections in the general population and intensive care COVID-19 bed occupancy rates are recorded in Germany. These data are statistically analyzed on a daily basis using epidemic SEIR (susceptible, exposed, infection, recovered) models using ordinary differential equations and multiple regression models.

**Results:**

Forecast results of the immediate trend (20-day forecast) of ICU occupancy by COVID-19 patients are made available to decision makers at various levels throughout the country.

**Conclusion:**

The forecasts are compared with the development of available ICU bed capacities in order to identify capacity limitations at an early stage and to enable short-term solutions to be made, such as supraregional transfers.

## Einleitung

Ein zentrales Element der Pandemiebewältigung und Grundlage zur datenbasierten Handlungssteuerung ist eine aktuelle, flächendeckende Erfassung und Vorhersage der unmittelbaren Entwicklung der Belegung durch intensivpflichtige COVID-19-Patienten*innen [1].

Um diesem Ziel Rechnung zu tragen, initiierte das Robert Koch-Institut (RKI) das Forschungsprojekt „Steuerungs-Prognose von intensivmedizinischen COVID-19-Kapazitäten“ (SPoCK).

In diesem Artikel werden Einblicke in die im Rahmen dieses Projekts entwickelten und fortlaufend weiterentwickelten statistisch-mathematischen Modelle gegeben.

**Figure 1:**
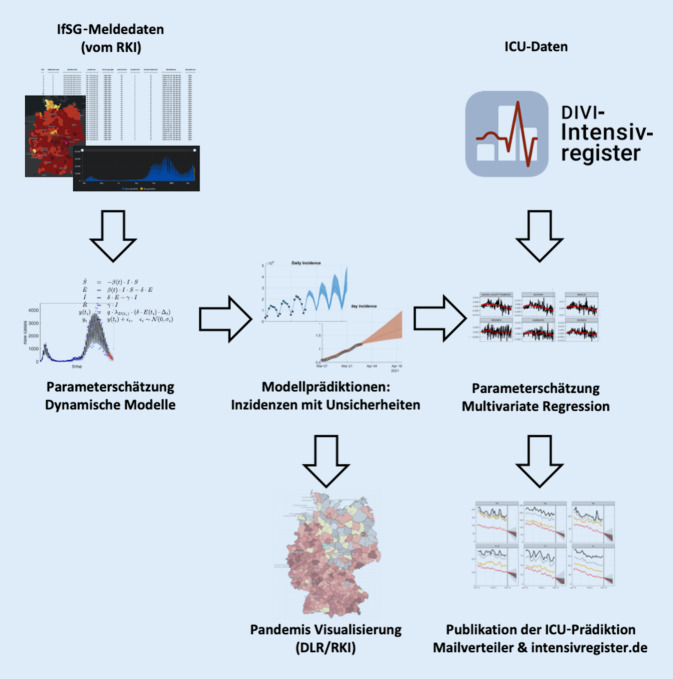

Die Abb. 1 zeigt die Struktur des Datenflusses und der Modellierung. In einem ersten Schritt werden die gemeldeten COVID-19-Neuinfektionen (gemäß den IfSG-Meldedaten) und deren wahrscheinlich tatsächlicher Verlauf mit einem dynamischen Modell geschätzt und Vorhersagen zur Entwicklung der COVID-19-Neuinfektionen generiert. Diese Vorhersagen werden im zweiten Schritt als ein Prädiktor für das Prognosemodell der zu erwartenden COVID-19-Intensivbettenbelegung benutzt. Die Prognoseergebnisse werden Entscheidungsträgern auf verschiedenen Ebenen zur Verfügung gestellt.

## Methoden

### Datengrundlage

Die Modellierung der intensivmedizinischen Bettenbelegung mit COVID-19-Patient*innen basiert primär auf 2 Datenquellen: 1) die durch das RKI täglich veröffentlichten Daten zu den inzidenten COVID-19-Neuinfektionen in der Bevölkerung (gemäß Meldeweg über die Gesundheitsämter) und 2) die täglich aktuellen Meldungen der intensivmedizinischen COVID-19-Belegung (prävalente Fälle) aus dem DIVI-Intensivregister, einem Kooperationsprojekt des RKI und der DIVI e. V. [2].

### Schritt 1: Modellierung und Prognose der SARS-CoV-2-Neuinfektionen in der Bevölkerung

Zunächst wird aus den täglich publizierten Meldedaten der Gesundheitsämter der Verlauf der COVID-19-Neuinfektionen geschätzt, wie er am wahrscheinlichsten tatsächlich verlaufen ist, sowie die Entwicklung geschätzt, wie sie sich wahrscheinlich in der nahen Zukunft fortsetzen wird. Unter Verwendung eines epidemischen SEIR-Modells aus gewöhnlichen Differenzialgleichungen werden u. a. die folgenden Faktoren berücksichtigt:

1. Schwankungen im Wochenverlauf, z. B. weniger Meldungen an Wochenenden;
2. Meldeverzug der Daten durch Übermittlung und Qualitätsprüfung;
3. regionale Unterschiede bez. Prävalenz und Dunkelziffer infizierter/erkrankter aber nicht gemeldeter Fälle;
4. Veränderungen der zeitabhängigen Ansteckungsrate, z. B. durch Impfungen, Virusmutationen sowie die Veranlassung und Akzeptanz politischer Eindämmungsmaßnahmen usw.

Bei dem gewählten Modellansatz [3] werden alle Parameter sowie deren Unsicherheit vollständig auf Grundlage der Daten, basierend auf den gemeldeten Inzidenzen, geschätzt. Die Meldungen der COVID-19-Neuinfektionen können mit einem Zählprozess verglichen werden, daher wird ein an die Poisson-Verteilung angelehntes Fehlermodell angenommen. Dies entspricht einer Normalverteilung mit einer Varianz proportional zur Anzahl neuer Fälle. Für die Prognose der zukünftigen Entwicklung der Neuinfektionen muss die zeitabhängige Infektionsrate extrapoliert werden. Da sich die Effekte der unter 4. genannten Faktoren nicht verlässlich vorhersagen lassen, wird der geschätzte Wert der Infektionsrate zum Zeitpunkt des letzten Datenpunkts fixiert, wobei dessen statistische Unsicherheit in die Vorhersageunsicherheit eingeht. Somit wird eine 20-Tage-Prognose unter der Annahme bzw. für ein Szenario erstellt, in dem sich die Infektionsrate durch die Effekte der unter 4. genannten Faktoren für den Prognosezeitraum nicht maßgeblich verändert. Zur Sicherstellung robuster Ergebnisse werden 2 unterschiedliche Modellansätze berechnet und zusammengeführt. Letztere unterscheiden sich in der Vorprozessierung der Meldedaten: Im ersten Ansatz werden die letzten beiden Tagesinzidenzen weggelassen, im zweiten Ansatz werden die Tagesinzidenzen mittels eines Nowcasting-Verfahren korrigiert. Beide Ansätze tragen also dem Meldeverzug Rechnung, der insbesondere die aktuellsten Tagesinzidenzen in Richtung kleiner Werte verzerrt.

Ein zentraler Aspekt ist auch, die Unsicherheiten der Vorhersagen anzugeben. Dazu wird neben der wahrscheinlichsten Trajektorie auch ein Intervall angegeben, indem sich der Verlauf mit einer gewissen (hier 95 %igen) Wahrscheinlichkeit befinden wird. Diese Vorhersageunsicherheit basiert in dem hier vorgestellten Ansatz auf den Unsicherheiten aller Modellparameter und wird mit der Profile-Likelihood-Methode [4] berechnet. Für die Kalibrierung der Modelle werden die Daten aus dem gesamten Zeitraum seit Beginn der Pandemie verwendet.

In diesem Modell werden insgesamt 26 Parameter geschätzt. Diese enthalten 18 dynamische Parameter: 14 Parameter für den Spline, der die zeitabhängige Infektionsrate approximiert, 3 Übergangsraten und einen Startwert der Dynamik. Weitere 7 Parameter beschreiben die Beobachtungsfunktion inklusive Wochenmodulation. Außerdem gibt es einen Fehlerparameter, der die Unsicherheit in den Beobachtungen widerspiegelt. Alle Parameter werden täglich neu geschätzt, was unter anderem deshalb nötig ist, weil sich die Stützstellen der zeitabhängigen Infektionsrate verschieben. Die tägliche Berechnung auf täglichen Inzidenzdaten seit 03. März 2020 führt am Analysetag 07. Juni 2021 zu 458 Datenpunkten, die für die Parameterschätzung benutzt werden. Für weitergehende und mathematische Details verweisen wir auf die zugehörige Veröffentlichung [3].

### Schritt 2: Modellierung und Prognose der COVID-19-Patient*innen in Intensivbehandlung

Im zweiten Schritt werden die Prognoseergebnisse aus Schritt 1 weiter für die Modellierung und Prognose der COVID-19-Patient*innen in Intensivbehandlung verwendet.

### Datenaufbereitung

Im DIVI-Intensivregister meldet jedes Akutkrankenhaus die tägliche aktuelle Anzahl der belegten Intensivbetten durch COVID-19-Patient*innen (prävalente Fälle). Durch die räumliche Auflösung können folgende Ebenen betrachtet werden: Versorgungscluster, Bundesländer, Kleeblätter (siehe Kleeblattregionen in Gräsner et al. [5]) und Deutschland.

### Modellierung

Für jede der verschiedenen o. g. räumlichen Ebenen werden separate und analoge Modelle gerechnet. Wir stellen den Ansatz exemplarisch anhand der Kleeblattebene vor.

Zuerst werden die täglichen Veränderungen der intensivmedizinischen (ITS-)COVID-Belegung zum Vortag durch das jeweilige Prävalenzverhältnis (Quotient der aktuellen Prävalenz zum Vortag) betrachtet. Das Hauptinteresse liegt hierbei im zeitlichen Trend der Prävalenzquotienten: Ein Trend größer als 1 bedeutet, dass zunehmend mehr Intensivbetten zur COVID-19-Behandlung benötigt werden. Mit diesem Vorgehen werden insbesondere Wendepunkte im zeitlichen Verlauf der intensivmedizinischen Bettenbelegung durch COVID-19-Patient*innen erkennbar.

Anschließend werden diese täglichen Veränderungen zum Vortag mithilfe linearer Regressionsmodelle in Abhängigkeit von mehreren Faktoren analysiert und modelliert. Zu den Hauptprädiktoren für die ITS-COVID-Belegung gehören der errechnete wahrscheinliche Verlauf und die zeitlichen Veränderungen der Neuinfektionen aus der Bevölkerung (Schritt 1). Die Datengrundlage weist ein durchschnittliches Zeitintervall von 5–7 Tagen zwischen einer COVID-19-Neuinfektion und der Aufnahme auf die Intensivstation auf. Infolgedessen wird die Anzahl der Neuinfektionen in der Bevölkerung als ein externer Prädiktor mit einem Zeitverzug von 5–7 Tagen für die Anzahl der COVID-19-Patient*innen in Intensivbehandlung in Korrelation gesetzt und repräsentiert den Außendruck auf die Intensivstationen. Weitere Prädiktoren sind u. a. die Altersverteilung in den Neuinfektionen basierend auf den Meldedaten der Gesundheitsämter. Weiter erlauben raum-zeitliche Interaktionsterme eine dynamische Flexibilität, d. h.: Die Effekte werden global mit allen Daten geschätzt, können aber lokal variieren. Für die Modellierung und Prognose der COVID-19-Patient*innen in Intensivbehandlung werden derzeit 20 Regressionskoeffizienten in dem Regressionsmodell auf Kleeblattebene geschätzt und verwendet.

#### Dynamische Schätzung und dynamische Prognose

Sowohl die IfSG-Meldedaten als auch die Fallzahlen des DIVI-Intensivregisters werden täglich aktualisiert. Die Modellierung wird gleichermaßen dynamisch aktualisiert, d. h.: Die Parameter und der Verlauf der COVID-19-Neuinfektionen aus Schritt 1 und die Regressionskoeffizienten aus Schritt 2 werden täglich neu geschätzt. Durch diese dynamische Schätzung können zugrunde liegende zeitlich-regionale Prozesse und Effekte (z. B. regionaler R‑Wert, Impfungen, Einfluss von Virusvarianten etc.) in Echtzeit indirekt über die Daten beobachtet, einbezogen und somit implizit gelernt werden (ohne dabei bestimmte externe Parameter festzulegen). Folglich werden alle Modellparameter ausschließlich basierend auf den Daten der 2 Systeme mit bundesweiter Vollabdeckung (siehe Datengrundlage) bestimmt. Aus der in Schritt 1 geschätzten Entwicklung der COVID-19-Neuinfektionen und den intensivmedizinischen Fallzahlen des DIVI-Intensivregisters wird dann die eigentliche 20-Tage-Prognose für den zu erwartenden Verlauf der Intensivbelegung mit COVID-19-Fällen berechnet. Dafür werden die Regressionskoeffizienten der jeweiligen Prädiktoren eingesetzt, wodurch man die Veränderung für die nächsten 20 Tage erhält.

Diese werden an den Wert der aktuellen Intensivbettenbelegung angeknüpft. Die prognostizierte Intensivbettenbelegung durch COVID-Patient*innen wird mit Prognoseintervallen graphisch dargestellt. Prognoseintervalle stellen einen Wertebereich dar, in dem zu einer gewissen Wahrscheinlichkeit die tatsächlichen Werte liegen. Somit ist es z. B. bei einem 95 %-Prognoseintervall sehr unwahrscheinlich (kleiner als 5 %), dass die tatsächliche Bettenbelegung außerhalb des Intervallbereichs (hier: hellgrauer Bereich) liegt.

## Ergebnisse

**Figure 2:**
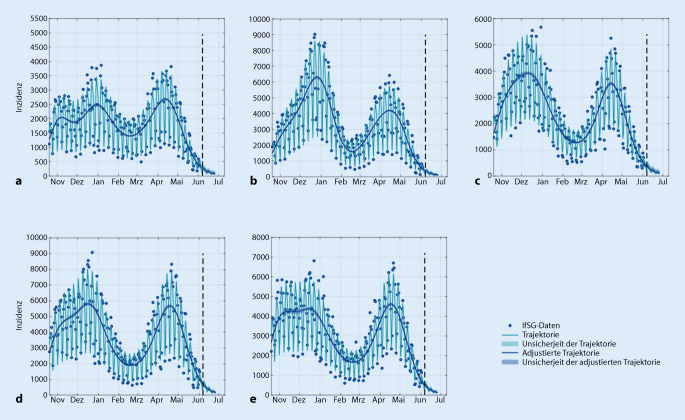

Das Prognosemodell fokussiert auf eine differenzierte Vorhersage für die zu erwartende Anzahl von intensivpflichtigen COVID-19-Patient*innen auf unterschiedlichen Versorgungsebenen (Deutschland, Kleeblätter [1], Bundesländer, Versorgungscluster) für maximal 20 Tage (Abb. 2 und 3). Des Weiteren werden die Prognosen der Entwicklung von betreibbaren intensivmedizinischen Bettenkapazitäten gegenübergestellt (Abb. 4), um frühzeitig Kapazitätsengpässe zu erkennen und kurzfristig reaktive Handlungssteuerungen, wie etwa überregionale Verlegungen, zu ermöglichen.

**Figure 3:**
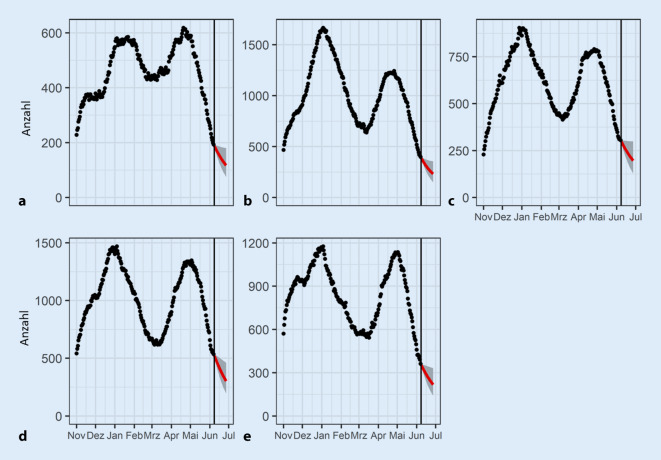

**Figure 4:**
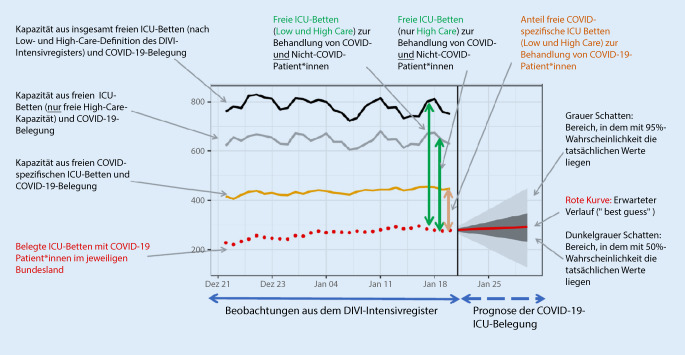

Somit orientieren sich die Prognosemodellierungen bewusst sehr nahe am aktuellen Lagebild. Die Prognosen verfolgen jedoch nicht das Ziel, den längerfristigen weiteren Pandemieverlauf vorherzusagen oder potenzielle Szenarien der Pandemieentwicklung über einen erweiterten Darstellungszeitraum abzubilden, um politische Entscheidungen zu unterstützen.

## Diskussion

Mit den hier beschriebenen Verfahren werden 2 wichtige Datenquellen (Meldungen der Gesundheitsämter und des DIVI-Intensivregisters) und ihre Einflussfaktoren sowie deren Zusammenspiel untereinander berücksichtigt.

Die Modelle werden regelmäßig auf Plausibilität und Prognoseleistung untersucht und mit parallel laufenden Alternativansätzen (z. B. Modellierung der Prävalenzdifferenz zum Vortag statt des Quotienten, bzw. direkte Modellierung der Prävalenz durch erweiterte Poisson-Regressionsmodelle) oder Erweiterungen (z. B. durch weitere Prädiktoren in den jeweiligen Regressionsmodellen) verglichen. Falls es zu einer Modelloptimierung kommt, werden unterschiedliche Modellansätze berechnet und zusammengeführt.

### Unterschiede zu anderen Prognoseansätzen

Die hier beschriebenen Methoden sind rein datenbasierte kurzzeitige Prognosen und unterscheiden sich somit von annahmebasierten Modellrechnungen, die vor allem verschiedene Handlungsoptionen aufzeigen sollen. Solche reinen Simulationsmodelle arbeiten meist ohne Primärdaten und sind stark abhängig von eingesetzten Parametern, z. B. aus Vorwissen oder der Literatur. Solche Ansätze eignen sich eher für die Untersuchung hypothetischer Szenarien z. B. zu erwarteten Effekten politischer Maßnahmen.

### Alternative Prognoseansätze zu Schritt 1

Es gibt zahlreiche Arbeiten, die mittels mathematischer Modellierung das Pandemiegeschehen prognostizieren. Auf dem European Forecast Hub [6] werden aktuell Prognosen von 23 Modellen oder Ensemble-Ansätzen für Deutschland veröffentlicht und wöchentlich aktualisiert (Stand 13.05.2021). Auf dem German and Polish COVID-19 ForecastHub [8] werden aktuell Prognosen von 17 Modellen oder Ensemble-Ansätzen für Deutschland veröffentlicht und wöchentlich aktualisiert ([7]; Stand 13.05.2021). Die deutschlandweit bekanntesten Ansätze sind unter anderem der COVID-19-Simulator [9] mit wöchentlichen Berichten der Universität Saarbrücken sowie verschiedene Onlinetools, bei denen unterschiedliche Parametrisierungen simuliert werden können.

Es sind dabei die jeweiligen Zielsetzungen in der Modellierung der Inzidenzen der Neuinfektionen zu unterscheiden: Simulationsstudien sind geeignet, unter verschiedenen Modellannahmen die Auswirkung von Maßnahmen zur Pandemiesteuerung zu untersuchen. Diese Resultate haben meist langfristigen und qualitativen Charakter und erlauben nur sehr eingeschränkt, die Ergebnisunsicherheiten zu quantifizieren. Im Gegensatz dazu versuchen Nowcasting und Forecasting, mit Methoden der Zeitreihenanalyse quantitative Aussagen über den aktuellen und den unmittelbar zu erwartenden Pandemieverlauf zu treffen. Für diese Zielsetzung sind auch agentenbasierte Modelle, stochastische Differenzialgleichungen (SDEs) und *Mixed-Effects-Modelle* denkbar.

### Alternative Prognoseansätze zu Schritt 2

Für die Prognose der COVID-19-Patient*innen in einer Intensivbehandlung gibt es einen sehr ähnlichen statistischen Ansatz von Farcomeni et al. [10], der ohne die raum-zeitliche Verknüpfung mit dem Außendruck auf Intensivstationen, d. h. die Anzahl der COVID-19-Neuinfektionen aus der Bevölkerung, auskommt. Der hier vorgestellte Ansatz erweitert prinzipiell den Ansatz von Farcomeni et al. [10] durch die Einbindung weiterer externer Prädiktoren.

Ein weiterer Ansatz ist das „*Bundesweites Belastungsmodell für Intensivstationen durch COVID-19*“ [11], das seinen Fokus vor allem auf den potenziellen Einfluss von hypothetischen Szenarien legt für einen zeitlich weiten Blick. Im Gegensatz dazu stützen sich die hier in dieser Arbeit beschriebenen Prognosemodelle primär auf die bisherige und aktuelle Situation, während keine Annahmen über die potenziell zukünftigen Veränderungen z. B. durch den Einfluss bevölkerungsweiter Maßnahmen getroffen werden. Eine Annahme für die Prognose ist, dass die aktuell vorliegende Entwicklung in dieser Form auch für die kommenden 20 Tage bestimmend ist, und fokussiert auf die unmittelbare Handlungssteuerung.

### Limitationen

Eine Hauptlimitation dieser Modelle liegt in der Natur der Daten (absolute Häufigkeiten im Zeitreihenformat). Dieses Datenformat erlaubt zwar Prognosen basierend auf populationsaggregierten Korrelationen, doch sie sind prinzipiell nicht für die Evaluationen zugrunde liegender Prozesse, wie z. B. veränderte Effekte bez. Impfungen, Altersverteilungen, Behandlungsmethoden, Virusvarianten, geeignet. Für entsprechend tiefergehende Studien sind patientenindividuelle Verlaufsdaten der gemeldeten SARS-CoV-2-Fälle und statistische Modelle sog. Multistadienmodelle notwendig [12, 13]. Dafür bedarf es einer entsprechenden Dateninfrastruktur, bei der Informationen der gemeldeten Neuinfektionsfälle aus der Bevölkerung mit individuellen Patientenverlaufsdaten sowie den Informationen der Behandlungskapazitäten der Krankenhäuser verknüpft sind.

Zusammenfassend lässt sich die Prognoseleistung wie folgt einordnen. Retrospektive Evaluationen zeigten, dass die kurzfristigen Prognosen (ca. 5–10 Tage) der COVID-19-Patient*innen in einer Intensivbehandlung relativ robust waren. Schwierig und herausfordernd sind sicherlich Wendepunkte, die zusätzlich noch regional unterschiedlich sind. Daher ist sowohl eine zeitnahe aktualisierte Modellierung und Schätzung als auch die Einbindung der Prognoseintervalle zur Einschätzung der Lage notwendig.

Schlussendlich sollte wie bei allen Prognosemodellen in der Infektionsepidemiologie auch hier das Prognoseparadoxon angesprochen werden: Wenn z. B. aufgrund der Prognosen überregionale Verlegungen oder politische Maßnahmen durchgeführt werden, kann die ursprüngliche Prognose nicht mehr wie vorhergesagt eintreten. Somit sollten die Annahmen, Stärken und Limitationen der zugrunde liegenden Prognosemodelle stets berücksichtigt werden, um die Vorhersagen differenziert bewerten zu können.
